# Malonylome of the plant growth promoting rhizobacterium with potent biocontrol activity, *Bacillus amyloliquefaciens* FZB42

**DOI:** 10.1016/j.dib.2016.12.029

**Published:** 2016-12-21

**Authors:** Ben Fan, Yu-Long Li, Lei Li, Xiao-Jun Peng, Chen Bu, Xiao-Qin Wu, Rainer Borriss

**Affiliations:** a Co-Innovation Center for Sustainable Forestry in Southern China, College of Forestry, Nanjing Forestry University, 210037 Nanjing, China; b RNA Biology Group, Institute for Molecular Infection Biology, University of Würzburg, 97080 Würzburg, Germany; c Jingjie PTM Biolabs (Hangzhou) Co. Ltd., Hangzhou 310018, China; d Fachgebiet Phytomedizin, Albrecht Daniel Thaer Institut für Agrar- und Gartenbauwissenschaften, Lebenswissenschaftliche Fakultät, Humboldt Universität zu Berlin, 14195 Berlin, Germany

## Abstract

The data presented in this article are related to the publication entitled “Malonylome analysis of rhizobacterium Bacillus amyloliquefaciens FZB42 reveals involvement of lysine malonylation in polyketide synthesis and plant–bacteria interactions”(doi:10.1016/j.jprot.2016.11.022) (B. Fan, Y. Li, L. Li et al.) [1]. This article presented the raw information of all malonyllysine sites identified by LC-MS/MS in the *Bacillus amyloliquefaciens* FZB42. Further, the functional features and conservation of the malonylated peptide/proteins were analyzed and made publicly available to enable critical or extended analyses.

**Specifications Table**

**Table:** 

Subject area	Biology
More specific subject area	Microbiology
Type of data	Tables
How data was acquired	mass spectroscopy and analysis
Data format	analyzed
Experimental factors	Trypic digestion, HPLC fractionation, enrichment by antibodies, LC-MS/MS
Experimental features	Proteins from *Bacillus amyloliquefaciens* FZB42 were digested and enriched for the peptides with malonylation sites which were finally identified by mass spectrometry.
Data source location	Nanjing, China
Data accessibility	with this article

**Value of the data**

1. *Bacillus amyloliquefaciens* subsp. *plantrum* is closely related with the model organism *Bacillus subtilis* and thus the data can be used to study malonylation of *B. subtilis*.
2. Our data provide a list of malonylated proteins, which are known to be involved in polyketide synthesis and plant-microbe interaction; researchers can use the data to study the roles of malonylation in polyketide synthesis and plant–microbe interaction.
3. The data allow crosstalk analysis of protein malonylation and other types of post-translational modification (PTM) in bacteria.
4. The data can be used for general analysis of protein features, functions, and regulations in *Bacillus* species.

## 1 Data

*Bacillus amyloliquefaciens* subsp. *plantrum* FZB42 is a representative of rhizobacteria with potent plant growth promoting and biocontrol activities [2], [3]. The global identification of post-translational malonylation on the proteins of *B. amyloliquefaciens* FZB42 has been performed [1]. The dataset of this article contains six tables (Tables 1–6) presenting the raw information and extended analysis of the malonylome of FZB42.

## 2 Experimental design, materials and methods

Proteins were extracted from the culture of *Bacillus amyloliquefaciens* FZB42 and digested by trypsin. The digested proteins were fractionated by HPLC before the peptides containing malonylation were enriched by anti-malonyllysine antibody beads. The enriched peptides were analyzed by LC-MS/MS for malonylation identification. Please see the publication “Malonylome analysis of rhizobacterium *Bacillus amyloliquefaciens* FZB42 reveals involvement of lysine malonylation in polyketide synthesis and plant-bacteria interactions”(doi:10.1016/j.jprot.2016.11.022) [1] for the details of Experimental Design, Materials and Methods.

## References

[1] B. Fan, Y. Li, L. Li, et al. Malonylome analysis of rhizobacterium *Bacillus amyloliquefaciens* FZB42 reveals involvement of lysine malonylation in polyketide synthesis and plant-bacteria interactions. J. Proteomics, 〈Doi:10.1016/j.jprot.2016.11.022〉 10.1016/j.jprot.2016.11.022 27939684

[2] Chen X., Koumoutsi A., Scholz R. (2007). Comparative analysis of the complete genome sequence of the plant growth-promoting bacterium *Bacillus amyloliquefaciens* FZB42. Nat. Biotechnol..

[3] Borriss R., Chen X., Rueckert C. (2011). Relationship of *Bacillus amyloliquefaciens* clades associated with strains DSM7T and FZB42: a proposal for *Bacillus amyloliquefaciens* subsp. *amyloliquefaciens* subsp. nov. and *Bacillus amyloliquefaciens* subsp. *plantarum* subsp. nov. based on their discriminating complete genome sequences. Int. J. Syst. Evol. Microbiol..

